# Elucidation of the effect of ionic liquid pretreatment on rice husk via structural analyses

**DOI:** 10.1186/1754-6834-5-67

**Published:** 2012-09-07

**Authors:** Teck Nam Ang, Gek Cheng Ngoh, Adeline Seak May Chua, Min Gyu Lee

**Affiliations:** 1 Department of Chemical Engineering, Faculty of Engineering, University of Malaya, 50603, Kuala Lumpur, Malaysia; 2 Division of Applied Chemical Engineering, Pukyong National University, Busan, 608-739, South Korea

**Keywords:** Rice husk, Ionic liquid, Dissolution, Pretreatment, Regenerated cellulose, Structural analysis

## Abstract

**Background:**

In the present study, three ionic liquids, namely 1-butyl-3-methylimidazolium chloride ([BMIM]Cl), 1-ethyl-3-methylimidazolium acetate ([EMIM]OAc), and 1-ethyl-3-methylimidazolium diethyl phosphate ([EMIM]DEP), were used to partially dissolve rice husk, after which the cellulose were regenerated by the addition of water. The aim of the investigation is to examine the implications of the ionic liquid pretreatments on rice husk composition and structure.

**Results:**

From the attenuated total reflectance Fourier transform-infrared (ATR FT-IR) spectroscopy, X-ray diffraction (XRD) and scanning electron microscopy (SEM) results, the regenerated cellulose were more amorphous, less crystalline, and possessed higher structural disruption compared with untreated rice husk. The major component of regenerated cellulose from [BMIM]Cl and [EMIM]DEP pretreatments was cellulose-rich material, while cellulose regenerated from [EMIM]OAc was a matrix of cellulose and lignin. Cellulose regenerated from ionic pretreatments could be saccharified via enzymatic hydrolysis, and resulted in relatively high reducing sugars yields, whereas enzymatic hydrolysis of untreated rice husk did not yield reducing sugars. Rice husk residues generated from the ionic liquid pretreatments had similar chemical composition and amorphousity to that of untreated rice husk, but with varying extent of surface disruption and swelling.

**Conclusions:**

The structural architecture of the regenerated cellulose and rice husk residues showed that they could be used for subsequent fermentation or derivation of cellulosic compounds. Therefore, ionic liquid pretreatment is an alternative in the pretreatment of lignocellulosic biomass in addition to the conventional chemical pretreatments.

## Background

The increasing demand for lignocellulosic feedstock derived from commodity crops, such as cotton plant and fiber wood crop, has prompted the need to prospect for alternative renewable resources, such as agricultural crop residues. These lignocellulosic crop residues are generated worldwide in vast amounts. In the Asia-Pacific region alone, approximately 1.2 billion tons of crop residues are generated annually [1]. These crop residues are inexpensive and are sustainable sources for biofuel production. Some of the potential lignocellulosic biomass used in bioconversion and their compositions are summarized in Table 1.

**Table 1:** Lignocellulosic biomass and their compositions

**Biomass**	**Content (%)**			**Reference**
	**Cellulose**	**Hemicellulose**	**Lignin**	
Alfalfa	33	18	8	[2]
Empty palm fruit bunch	60	22	18	[3]
Rice straw	36	17	23	[4]
Sugarcane bagasse	41	30	21	[5]
Sweet sorghum	36	18	16	[6]
Wheat straw	49	28	8	[7]

However, enzymatic saccharification of these untreated crop residues leads to low reducing sugar yields. Hence, pretreatment that disrupts the recalcitrant lignocellulosic biomass is necessary to enhance the saccharification of cellulose/hemicellulose into reducing sugars. Physical, chemical and a combination of physical/chemical pretreatments are the commonly employed methods in pretreating lignocellulosic biomass. Some of these methods require long residence times, high energy consumption, and carry the risk of sugar degradation when pretreatment is conducted at high temperatures [8,9]. In consideration of these shortcomings, continual efforts have been invested to explore alternative pretreatments, one of which is via the application of green solvent - ionic liquids that is reported in this study.

Ionic liquids with cellulose-dissolving ability offer a novel solution for pretreating lignocellulosic biomass [10]. Various ionic liquids, such as 1-butyl-3-methylimidazolium chloride ([BMIM][Cl]) [11], 1-ethyl-3-methylimidazolium acetate ([EMIM][OAc]) [12], and 1-ethyl-3-methylimidazolium diethyl phosphate ([EMIM][DEP]) [13], have been applied as solvents in pretreatment step before enzymatic saccharification to enhance reducing sugars yield. Lignocellulosic biomass pretreated with ionic liquid is favorable for subsequent enzymatic hydrolysis due to their reduced cellulose crystallinity and decreased lignin content [11,13-15]. Li et al. [12] reported a significant improvement of reducing sugars yield of 17-fold from enzymatic saccharification of ionic liquid-pretreated switchgrass. In another study, enzymatic hydrolysis of ionic liquid-pretreated forest residues too exhibited increment in reducing sugars yield [16]. Besides the significant improvement of yield, ionic liquids are greener pretreatment media because they can be recycled and reused in the dissolution process [17].

Rice husk (*Oryza sativa*) is one of the lignocellulosic residues that have attracted much attention among researchers due to its relatively high cellulose content and its potential to be used in biofuel production. The effects of acid and alkaline pretreatments on rice husk have previously been reported by Ang et al. [18]. However, the application of ionic liquid pretreatments on rice husk has not been reported elsewhere. To gain a greater insight into ionic liquid pretreatment, the effect of ionic liquids with different anionic groups on the structural changes of rice husk was investigated. The structural architecture studies were conducted on both the regenerated cellulose and the rice husk residues from ionic liquid pretreatments.

## Results and discussion

### Dissolution of rice husk

In this study, the rice husk sample contained 53.18 ± 0.44% (w/w) cellulose, 4.63 ± 0.58% (w/w) hemicellulose and 19.67 ± 0.28% (w/w) lignin [19]. Rice husk is often used in “low value for money” applications; for instance, it is either disposed as waste or burnt as fuel [20,21]. Sometimes it is used as a low-cost filler in animal feeds [22] or as fertilizer [23]. By applying an appropriate pretreatment, rice husk with relatively high cellulose content could be an attractive source for saccharification or derivatization into other useful products.

The effects of three ionic liquids ([BMIM]Cl, [EMIM]DEP and [EMIM]OAc) on the dissolution of rice husk and subsequent regeneration of cellulose were investigated. Previous study showed that prolong pretreatment at high temperatures possesses the risk of degrading the dissolved cellulose [24]. Thus, the current experimental conditions (heating at 100°C for 10 hours) were selected as a compromise to allow sufficient cellulose dissolution, while minimizing the possibility of cellulose degradation and also reducing energy for pretreatment.

**Figure 1:**
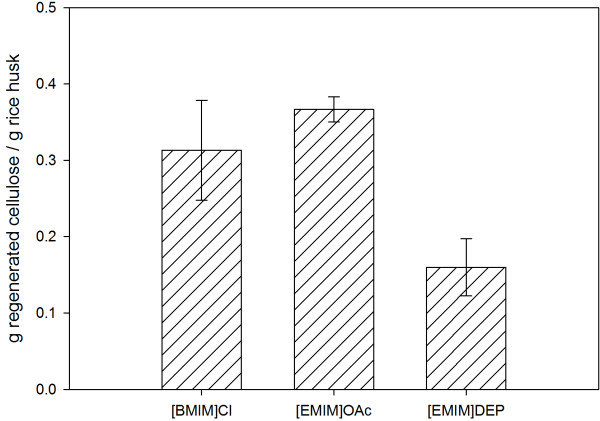
Cellulose regenerated from ionic liquid pretreatments.

From the observation, all the ionic liquids investigated did not completely dissolve rice husk. The amount of cellulose dissolved and subsequently regenerated from the respective ionic liquids is shown in Figure 1. After 10 hours of heating, the acetate-based ionic liquid [EMIM]OAc and chloride-based ionic liquid [BMIM]Cl produced 0.37 and 0.31 g regenerated cellulose/g rice husk, respectively. Under the same pretreatment conditions, [EMIM]DEP gave about 0.16 g regenerated cellulose/g rice husk. All the reaction mixtures have dark brown appearance after the dissolution process. The color was imparted by the dissolved lignin of lignocellulosic matrix [13,15,24,25]. Dissolution of rice husk is influenced by the interactions between anion of the ionic liquid and hydroxyl group of the cellulose [11,15,26]. Anion of the ionic liquid acts as the hydrogen-bond acceptor in dissolution where it interacts specifically with the hydroxyl protons of the cellulosic materials [27,28] and facilitates the formation of hydrogen bonds between cellulose and ionic liquid. Among the three ionic liquids, the acetate-based [EMIM]OAc has higher hydrogen-bond basicity [29], which explains its better dissolubility than [BMIM]Cl and [EMIM]DEP.

On the other hand, rice husk residues separated from the reaction mixtures were swollen compared to the untreated rice husk. This is due to the diffusion of the ionic liquids into the rice husk matrix that subsequently facilitates the dissolution of the rice husk [30]. Among the ionic liquids examined, rice husk residue from the [EMIM]OAc pretreatment were the most severely swollen, whereas [BMIM]Cl and [EMIM]DEP did not show extensive swelling.

### Structural characterization

#### ATR FT-IR analysis

**Figure 2:**
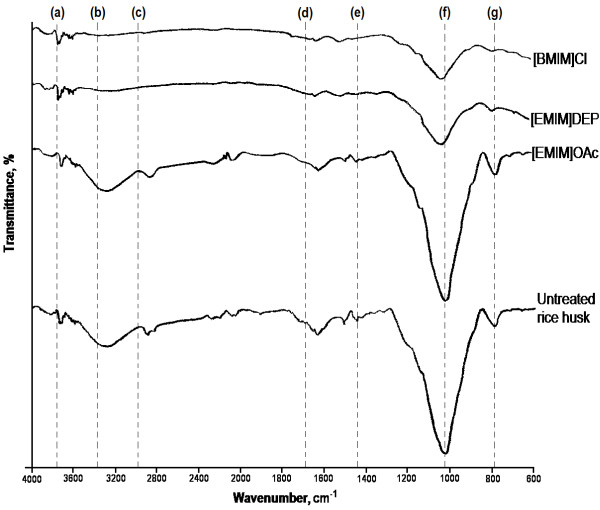
**ATR FT-IR spectra of regenerated cellulose and untreated rice husk.** FT-IR spectral bands (cm^-1^): (**a**) 3750; (**b**) 3312; (**c**) 2919; (**d**) 1637; (**e**) 1457; (**f**) 1035; (**g**) 798.

In order to gain more insights into the effects of ionic liquid pretreatments, studies on the chemical and structural characteristics of the regenerated cellulose and rice husk residue are essential. The regenerated cellulose from all the ionic liquids had altered chemical and structural characteristics compared to the untreated rice husk. The structural changes of regenerated cellulose were analyzed by ATR FT-IR spectroscopy in the region of 600 – 4000 cm^-1^, which is commonly used to study the fine structural characteristics of cellulose [31,32]. The spectra of regenerated cellulose from the ionic liquids and untreated rice husk are presented in Figure 2. The absorption bands at 798, 1035, 1457, 1513, 1637, 2919, 3312, and 3750 cm^-1^ in the spectrum of untreated rice husk are associated with native rice husk lignocelluloses. Table 2 shows the group frequency of absorption bands of untreated rice husk and their assignments. Both cellulose/hemicellulose- and lignin-associated bands are present in the spectrum of untreated rice husk, and this suggests the presence of lignin-carbohydrate matrix in rice husk. Spectra of all the regenerated cellulose show the strongest absorption band at about 1035 cm^-1^. This band corresponds to the C-O stretching vibration in both cellulose/hemicellulose and lignin, and it explains the lignocellulosic nature of rice husk [31,33,34].

**Table 2:** Group frequency of absorption bands of rice husk and regenerated cellulose

**Group frequency, wavenumber, cm**^**-1**^	**Origin**	**Assignment**	**Reference**
800 – 950	C-H	C-H deformation vibration in cellulose	[34]
~1035	C-O	C-O stretching vibration in cellulose/hemicellulose and aryl-OH group in lignin	[31]
			[33]
			[34]
~1457	C-H	Asymmetric bending of CH_3_ and methoxy (-OCH_3_) groups present in lignin	[34]
			[33]
			[24]
~1513	C = C-C^*a*^	Aromatic skeletal stretching in lignin	[35]
			[36]
~1637	O-H	O-H bending vibration of adsorbed water molecules	[31]
			[37]
~2919	C-H	C-H stretching in cellulose-rich material	[38]
			[39]
2995 – 4000	O-H	Free and hydrogen-bonded OH stretching	[40]
			[31]
			[38]

^*a*^ C = C – C is used as an approximation of the aromatic skeleton.

[EMIM]OAc showed the highest dissolution of rice husk (Section ‘Dissolution of rice husk’), and its regenerated cellulose possessed all the absorption bands present in the untreated rice husk. This clearly suggests that [EMIM]OAc does not selectively dissolve cellulose, but both cellulose and lignin in the rice husk lignocellulosic matrix. This ionic liquid has been reported to be capable of dissolving cellulose and lignin [41], and various lignocellulosic biomass [24,41,42]. Furthermore, [EMIM]OAc-treated cellulose showed higher intensity at band 797 cm^-1^, indicating that the regenerated cellulose was more amorphous than the untreated rice husk. The band at about 800 cm^-1^ is sensitive to the amount of amorphous cellulose present in the regenerated material, where broadening of this band indicates higher amorphousity of the regenerated cellulose. The dissolution and subsequent regeneration of the hemicellulose fraction might contribute to higher degree of amorphousity of the regenerated cellulose.

In comparison, the spectra of regenerated cellulose from [BMIM]Cl and [EMIM]DEP dissolution were different from the spectrum of the untreated rice husk, where some absorption bands were absent. In the spectra of regenerated cellulose of these two ionic liquid pretreatments, the band in the region of 800 – 950 cm^-1^ is broader implying a higher amount of disordered cellulosic structure [37,38]. The disorder of cellulosic structure is very likely caused by the deformation vibration of β-glycosidic linkages and hydrogen bond rearrangement [34,37]. In addition, [BMIM]Cl- and [EMIM]DEP-regenerated cellulose exhibited reduced absorbance at 1035 cm^-1^, which might have resulted from the degradation of cellulose/hemicellulose during heating. The degradation shortens cellulose chains leading to the reduction in C-O-C pyranose ring skeletal stretching [39]. Moreover, the degradation of cellulose also reduced C-H stretching at 2896 cm^-1^ and free/hydrogen-bonded OH stretching at 3312 cm^-1^ of these regenerated cellulose. The disappearance of absorption band at 1457 cm^-1^ suggests the removal of lignin in regenerated cellulose of [BMIM]Cl and [EMIM]DEP.

**Figure 3:**
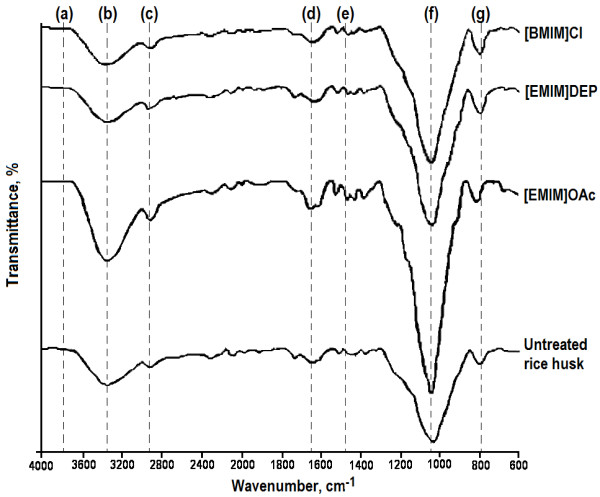
**ATR FT-IR spectra of rice husk residues and untreated rice husk.** FT-IR spectral bands (cm^-1^): (**a**) 3750; (**b**) 3312; (**c**) 2919; (**d**) 1637; (**e**) 1457; (**f**) 1035; (**g**) 798.

The spectra of rice husk residues after ionic liquid dissolution were also recorded using ATR FT-IR (Figure 3). All the absorption bands that occurred in the untreated rice husk were present in the spectra of the rice husk residues, indicating that both have similar compositions. An obvious change in intensity was observed in the band of approximately 1035 cm^-1^. The transmittance of this band increased in all the rice husk residues compared to their untreated counter-parts. This indicates that the rice husk residues contained considerable amounts of cellulose/hemicellulose, and possibly lignin, after the ionic liquid pretreatments. Besides, this also implies that ionic liquid pretreatment might dissolve components other than cellulose/hemicellulose, whereby the relative cellulose/hemicellulose content of rice husk residues increases proportionately. The intensity of absorption band in the region 800 – 950 cm^-1^ remains unchanged, signifying that both the rice husk residues and untreated rice husk do not vary very much in terms of amorphousity.

#### XRD analysis

The crystallinity of the rice husk samples was analyzed by XRD. A less crystalline regenerated cellulose structures was confirmed by XRD analysis with the occurrence of a sharper peak at 2*θ* = 18.7° compared with the untreated rice husk [43]. The lower crystallinity index indicates a higher amount of amorphous cellulose present in the regenerated cellulose [44]. All the regenerated cellulose has an estimated of 25% lower crystallinity index compared to the untreated rice husk (Table 3). Among the regenerated cellulose, cellulose regenerated from [EMIM]DEP pretreatment gave the lowest crystallinity index (32.0), followed by the regenerated cellulose of [EMIM]OAc and [BMIM]Cl pretreatments, which were 34.4 and 37.7, respectively. The results complemented and confirmed the findings of ATR FT-IR analysis reported previously, in which the cellulose regenerated from ionic liquid pretreatments exhibited higher amorphousity.

**Table 3:** Crystallinity indexes of untreated rice husk, regenerated cellulose and rice husk residue

**Rice husk sample**	***Crystallinity index***
Untreated rice husk	46.0
Regenerated cellulose ([BMIM]Cl)	37.7
Regenerated cellulose ([EMIM]OAc)	34.4
Regenerated cellulose ([EMIM]DEP)	32.0
Rice husk residue ([BMIM]Cl)	56.1
Rice husk residue ([EMIM]OAc)	39.1
Rice husk residue ([EMIM]DEP)	49.5

Rice husk residues of [BMIM]Cl and [EMIM]DEP pretreatments showed higher crystallinity index compared with the untreated rice husk; rice husk residue of [EMIM]OAc pretreatment showed slightly lower crystallinity index that is comparable to the untreated rice husk (Table 3). The dissolution of amorphous cellulose/hemicellulose of rice husk in ionic liquid, leaving the more crystalline lignocellulosic matrix in the residue, might be the main cause of the higher crystallinity index in rice husk residues of both [BMIM]Cl and [EMIM]DEP pretreatments. In contrast, the lower crystallinity of rice husk residue of [EMIM]OAc pretreatment might be due to the action of the ionic liquid that causes swelling to the structure of rice husk.

#### SEM analysis

**Figure 4:**
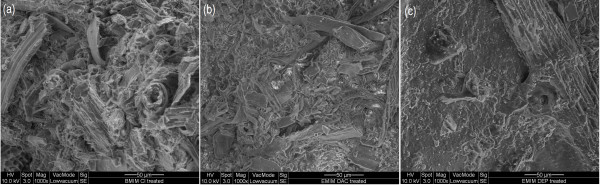
SEM images of regenerated cellulose from (a) [BMIM]Cl, (b) [EMIM]OAc, and (c) [EMIM]DEP pretreatments.

The structural morphology of cellulose regenerated from the ionic liquids was examined by SEM (Figure 4). All the regenerated cellulose showed rough and conglomerate textures [14] and these ribbon-like fiber aggregates were disorderly arranged in the matrix. The images of SEM were in agreement with the findings of ATR FT-IR; the organized structure commonly present in native lignocellulosic biomass was absent [45], signifying that the structure of the regenerated cellulose was more amorphous. This also indirectly indicates that, with ionic liquid pretreatment, crystallinity of the cellulose could be reduced compared to the untreated rice husk.

**Figure 5:**
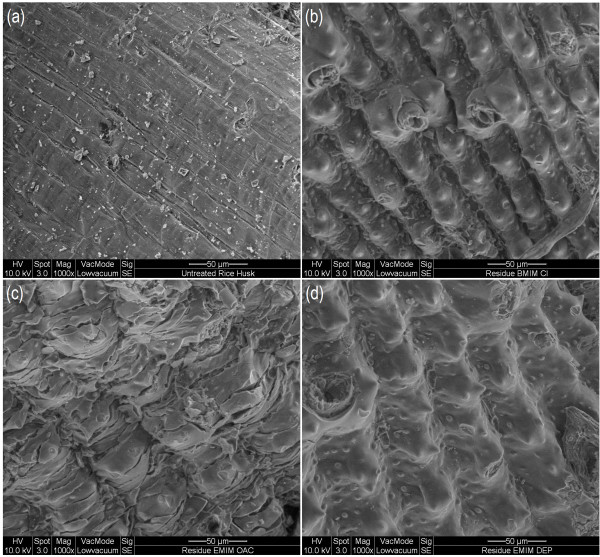
SEM images of (a) untreated rice husk and rice husk residues of (b) [BMIM]Cl, (c) [EMIM]OAc, and (d) [EMIM]DEP pretreatments.

The SEM images of the rice husk residues are shown in Figure 5. Although the residues did not show much changes in its lignocellulosic composition compared to the untreated rice husk in the ATR FT-IR analysis, the SEM images show that the surface structure of the rice husk residues changed significantly. The untreated rice husk was intact and had a rather smooth surface (Figure 5a) while the surface of rice husk residues appeared to be uneven and had cracks (Figure 5b, 5c and 5d). Rice husk residue from [EMIM]OAc-pretreatment was the most severely disrupted followed by [BMIM]Cl- and [EMIM]DEP-pretreatments. The disruption of the residue surface might have been caused by the solvating action of the ionic liquids, in which the outer lignocellulosic matrix of rice husk was swelled and dissolved in the ionic liquids. Rice husk residue from [EMIM]OAc pretreatment demonstrated the most severe swelling on the surface structure (Figure 5c) and this pretreatment also gave the highest amount of regenerated cellulose in the dissolution and regeneration study (Section ‘Dissolution of rice husk’). This indicates that the dissolution and subsequent regeneration of cellulose depend on the degree of swelling on the biomass. The dissolution process was preceded by extensive swelling of the rice husk matrix, which could be observed from the swollen appearance of the rice husk residues.

#### Comparison of ionic liquid pretreatments

The quantitative yield of regenerated cellulose reported in ‘Dissolution of rice husk’ indicates only the efficiency of the ionic liquids in dissolving rice husk. Information on structural characterization of the regenerated cellulose as well as the rice husk residues is helpful in the selection of a suitable ionic liquid pretreatment for lignocellulosic biomass. The ATR FT-IR and SEM analyses suggested that regenerated cellulose of the ionic liquid pretreatments comprise of cellulose-rich materials, which were more amorphous compared to the untreated rice husk. According to the XRD analysis, regenerated cellulose of the ionic liquid pretreatments had comparable crystallinity, with cellulose regenerated from [EMIM]DEP and [EMIM]OAc pretreatments showing a lower crystallinity.

Apart from the regenerated cellulose, rice husk residues from the ionic liquid pretreatments could be potential substrates for bioconversion into valuable compounds. The disrupted surface structure of the rice husk residues were favorable for solid-state fermentation, where it facilitates microbial growth by allowing access of microbes to the lignocellulosic matrix. SEM investigation demonstrated surface structure disruption of the rice husk residues after ionic liquid pretreatments, whereby [EMIM]OAc-treated rice husk residue showed the highest degree of structural disruption, followed by rice husk residues of [BMIM]Cl and [EMIM]DEP pretreatments. Besides, rice husk residue of [EMIM]OAc was found to have lower crystallinity after pretreatment compared to the other two ionic liquids. Nonetheless, chemical compositions of the rice husk residues remain relatively the same as those of the untreated rice husk.

The findings of structural characterization suggested that regenerated cellulose of [EMIM]OAc is amorphous and has low crystallinity, whereas its rice husk residue showed rigorously disrupted structure with reduced crystallinity. Therefore, [EMIM]OAc is a potential ionic liquid for the pretreatment of rice husk.

#### Enzymatic hydrolysis of regenerated cellulose and untreated rice husk

**Figure 6:**
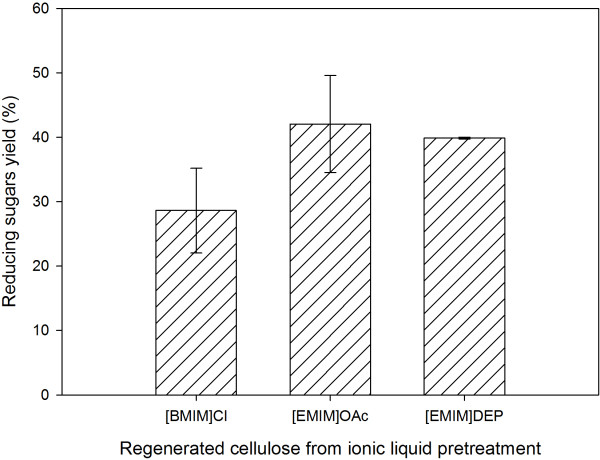
**Reducing sugars yields of cellulose regenerated from different ionic liquids.** Enzymatic hydrolysis conditions: substrate consistency, 2%; cellulase activity, 50 FPU/g regenerated cellulose; incubation, 50°C for 48 hours.

To confirm the digestibility of the regenerated cellulose from ionic liquid pretreatment, enzyme hydrolysis of the regenerated cellulose were carried out. Figure 6 shows the reducing sugars yields of the regenerated cellulose after enzymatic hydrolysis. Among the ionic liquids investigated, [EMIM]OAc-regenerated cellulose possessed the highest reducing sugars yield (42.1%) followed by [EMIM]DEP (39.9%) and [BMIM]Cl (28.6%). The higher yield from enzymatic hydrolysis of [EMIM]OAc-regenerated cellulose was in line with the observations of structural analysis which indicated a more disrupted and amorphous structure. Although [EMIM]DEP dissolves rice husk the least among the ionic liquids studied, cellulose regenerated from [EMIM]DEP pretreatment has better digestibility than [BMIM]Cl, which is well reported for its cellulose dissolution ability [14]. The better digestibility demonstrated by [EMIM]DEP might be due to its ability to delignify rice husk. It was also found that enzymatic hydrolysis of untreated rice husk did not produce detectable reducing sugars.

## Conclusions

Besides acid and alkaline pretreatments, ionic liquid pretreatment can be used for pretreating lignocellulosic biomass. This study found that the chemical composition of the regenerated cellulose varies with the type of ionic liquid used. The ionic liquids [BMIM]Cl and [EMIM]DEP delignified the lignocellulosic rice husk, indicating their potential to be used in producing regenerated cellulose for enzymatic saccharification or cellulose derivatives. On the other hand, [EMIM]OAc dissolved the entire lignocelluloses and imparted surface structure disruption on the regenerated cellulose that is desired for subsequent fermentation or derivation of cellulosic compounds. The regenerated cellulose were more amorphous and had lower crystallinity compared with the untreated rice husk, whereas the rice husk residues showed a certain degree of structural disruption. The study also demonstrated that enzymatic hydrolysis of the regenerated cellulose resulted in higher yield compared to the untreated rice husk. Both the regenerated cellulose and rice husk residue revealed desirable structural changes in this study, which suggested that ionic liquid pretreatment is beneficial for conversion into value-added products. It is also essential to select a suitable ionic liquid pretreatment depending on the final application of the regenerated cellulose and rice husk residue.

## Methods

### Material and reagents

Rice husk samples were obtained from Selangor, Malaysia. The rice husk was first washed and dried before being ground to approximately 30 mesh sizes (500 μm). Ground rice husk samples were stored in a dry cabinet prior to use.

**Figure 7:**
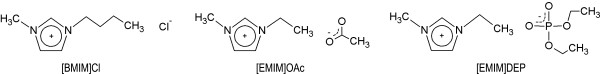
Chemical structures of the ionic liquids.

The ionic liquid 1-butyl-3-methylimidazolium chloride ([BMIM]Cl) was purchased from Merck (Germany), while 1-ethyl-3-methylimidazolium acetate ([EMIM]OAc) and 1-ethyl-3-methylimidazolium diethyl phosphate ([EMIM]DEP) were purchased from Sigma-Aldrich (USA). The ionic liquids were used without further purification. Their chemical structures are illustrated in Figure 7.

Cellulase from *Trichoderma viride* (Cellulase Onozuka R-10, catalogue # 102321) was purchased from Merck (Germany). The CMC activity of the Cellulase Onozuka R-10 was reported to be ≥ 1 U/mg. The IUPAC Filter Paper Assay was determined according to the procedure outlined by Ghose [46]. All the chemicals and reagents used were of analytical grade.

### Dissolution of rice husk

In ionic liquid pretreatment, a rice husk-ionic liquid mixture in a ratio of 1.5% (w/v) was heated to 100°C and pretreated for 10 hours in a block heater (HACH DRB200, USA). At the end of the pretreatment, the reaction mixture consisted of ionic liquid-dissolved cellulose and undissolved rice husk (hereafter called rice husk residue). The dissolution of rice husk was carried out in triplicate.

### Cellulose regeneration and residue separation

After the dissolution, an equal volume of deionised water (Sartorius, arium® 611UF, Germany) was added to the clear reaction mixture to precipitate regenerated cellulose before the rice husk residue was filtered according to the procedure as outlined by Ang et al. [19]. The cellulose-rich material (henceforth called regenerated cellulose) precipitated from the mixture was filtered. Both the regenerated cellulose and rice husk residue were washed with deionised water to remove the ionic liquid completely, and dried in an oven at 60°C prior to analyses.

### Structural characterization

#### ATR FT-IR analysis

The ATR FT-IR spectra of the samples between 600 and 4000 cm^-1^ at 4 cm^-1^ nominal resolution at room temperature were recorded using a FT-IR/FT-FIR spectrometer (Perkin Elmer, Spectrum 400, USA). The spectra are presented as relative transmittance percentage (%) of wave number (cm^-1^) and their background was recorded with an empty cell.

#### XRD analysis

XRD diffractogram of the rice husk samples was acquired with D8 Advance X-Ray Diffractometer (Bruker AXS, USA). The samples were scanned in the range of 10 – 80° (2*θ*) with a step size of 0.02° and step time of 1 s at 40 kV and 40 mA under ambient temperature. Crystallinity index (*CrI*) of the rice husk samples was computed by using Equation (1) [47].

(1) $$CrI=\frac{I_{002}-I_{\text{am}}}{I_{002}}\times 100$$

where *I*_002_ = maximum intensity of crystalline portion in rice husk sample at about 2*θ* = 22.6°, *I*_am_ = intensity attributed to the amorphous portion of rice husk sample at 2*θ* = ~18.7°.

#### SEM analysis

SEM images were obtained with a Quanta 200 FESEM (FEI, USA) scanning electron microscope operated at 10 kV accelerating voltage. The samples obtained in the dissolution of rice husk and regeneration of cellulose were affixed onto aluminum stubs with double sided adhesive carbon tapes and examined without metal-coating under low vacuum mode.

#### Enzymatic hydrolysis

Regenerated cellulose from the ionic liquid pretreatments was hydrolyzed using Cellulase Onozuka R-10 with a loading of 50 FPU/g substrate. Enzymatic hydrolysis of rice husk samples was carried out in 50 mM acetate buffer solution (pH 4.8) at 50°C for 48 h [5]. After the reaction, the samples were centrifuged at 10,000 *g* for 3 minutes. The concentration of total reducing sugars in supernatant was determined via DNS method [46,48]. The total reducing sugars yield obtained from enzymatic hydrolysis was computed according to Li et al. [13]. All the experiments were conducted in duplicate.

## Abbreviations

[BMIM]Cl: 1-butyl-3-methylimidazolium chloride; [EMIM]DEP: 1-ethyl-3-methylimidazolium diethyl phosphate; [EMIM]OAc: 1-ethyl-3-methylimidazolium acetate; ATR FT-IR: Attenuated total reflectance Fourier transform-infrared; SEM: Scanning electron microscopy; XRD: X-ray diffraction.

## Competing interests

The authors declare that they have no competing interests.

## Authors’ contributions

GCN, ASMC, and MGL (supervisors) conceived the study. TNA carried out the pretreatment experiments and analyses, and drafted the manuscript. GCN and ASMC participated in the test design and supervision, and helped to draft the manuscript. MGL provided ideas on the methodology and proofread the manuscript. All authors read and approved the final manuscript.
